# The role of hypoxia in the tumor microenvironment and development of cancer stem cell: a novel approach to developing treatment

**DOI:** 10.1186/s12935-020-01719-5

**Published:** 2021-01-20

**Authors:** Asieh Emami Nejad, Simin Najafgholian, Alireza Rostami, Alireza Sistani, Samaneh Shojaeifar, Mojgan Esparvarinha, Reza Nedaeinia, Shaghayegh Haghjooy Javanmard, Marjan Taherian, Mojtaba Ahmadlou, Rasoul Salehi, Bahman Sadeghi, Mostafa Manian

**Affiliations:** 1 Department of Biology, Payame Noor University (PNU), P.O.Box 19395-3697, Tehran, Iran; 2 Department of Emergency Medicine, School of Medicine , Arak University of Medical Sciences, Arak, Iran; 3 Department of Surgery, School of Medicine Amiralmomenin Hospital, Arak University of Medical Sciences, Arak, Iran; 4 Department of Emergency Medicine, School of Medicine Valiasr Hospital, Arak University of Medical Sciences, Arak, Iran; 5 Department of Midwifery, Faculty of Nursing and Midwifery , Arak University of Medical Sciences , Arak, Iran; 6 Department of Immunology, School of Medicine , Tabriz University of Medical Sciences , Tabriz, Iran; 7 Pediatric Inherited Diseases Research Center, Research Institute for Primordial Prevention of Non-Communicable Disease , Isfahan University of Medical Sciences , Isfahan, Iran; 8 Applied Physiology Research Center, Cardiovascular Research Institute, Isfahan University of Medical Sciences , Isfahan, Iran; 9 Department of Immunology, School of Medicine, Iran University of Medical Sciences, Tehran, Iran; 10 Sciences Medical of University Arak, Hospital Amiralmomenin, Center Development Research Clinical, Arak, Iran; 11 Department of Genetics and Molecular Biology, School of Medicine , Isfahan University of Medical Sciences , Isfahan, Iran; 12 Department of Health and Community Medicine, School of Medicine, Arak University of Medical Sciences, Arak, 3848176341 Iran; 13 Department of Medical Laboratory Science, Faculty of Medical Science Kermanshah Branch, Islamic Azad University, Imam Khomeini Campus, Farhikhtegan Bld., Shahid J’afari St., Kermanshah, 3848176341 Iran

**Keywords:** Hypoxia, Tumor microenvironment, Cancer progression, Cancer stem cells, Tumor treatment

## Abstract

Hypoxia is a common feature of solid tumors, and develops because of the rapid growth of the tumor that outstrips the oxygen supply, and impaired blood flow due to the formation of abnormal blood vessels supplying the tumor. It has been reported that tumor hypoxia can: activate angiogenesis, thereby enhancing invasiveness and risk of metastasis; increase survival of tumor, as well as suppress anti-tumor immunity and hamper the therapeutic response. Hypoxia mediates these effects by several potential mechanisms: altering gene expression, the activation of oncogenes, inactivation of suppressor genes, reducing genomic stability and clonal selection. We have reviewed the effects of hypoxia on tumor biology and the possible strategiesto manage the hypoxic tumor microenvironment (TME), highlighting the potential use of cancer stem cells in tumor treatment.

## Background

Solid tumors are the most common forms of cancer, and they account for high levels of morbidity and mortality globally [1]. They consist of an abnormal mass of cells containing blood and lymphatic vessels, components of the extracellular matrix (ECM), heterogeneous cells populations including fibroblasts, cancer stem cells (CSCs) and immune cells [2]. Solid tumors are commonly affected by hypoxia. In the tumor microenvironment (TME), uncontrolled cell proliferation often exceeds the ability to satisfy the oxygen demand from the preexisting blood vessels. This usually occurs when the tumor exceeds a diameter of approximately 1 mm [3, 4]. Tumor hypoxia-induced responses, include: altered gene expression, suppressing apoptosis, or promoting autophagy [5, 6], stimulation of the epithelial-mesenchymal transition (EMT), malignant progression and distant tumor metastasis [7, 8], enhanced angiogenesis and vasculogenesis [9–11], and changes in anabolic phenotype to core cellular metabolism [12, 13]. Moreover, hypoxia is also implicated in genomic instability due to the increased production of reactive oxygen species (ROS) and alterations in the DNA damage repair pathways [14, 15]. Hypoxia also enhances the aggressiveness of tumors by clonal selection. The new and more invasive selected clones lead to a vicious cycle of hypoxia, that act as a barrier to conventional cancer therapy, including radiotherapy, chemotherapy and phototherapy [16]. Hypoxia also impacts on the immune system through different pathways and contributes to a reduced anti-tumor response [16, 17]. Furthermore, there is mounting evidence suggesting that CSCs that are affected by hypoxia are largely responsible for tumor resistance and recurrence after conventional therapy. In contrast, there is also data in the literature that suggest that hypoxia makes tumor cells more sensitive to chemotherapy. However, it is generally accepted that tumors that are hypoxic are associated with a poorer outcome. In this review, we describe the events in the tumor milieu that are influenced by hypoxia and lead to tumor expansion and malignant progression favoring immune escape, frustrated anti-tumor therapy, and eventually tumor relapse, highlighting the function of hypoxic CSCs. Specifically, we will pursue the following: (i) the main transcription factors in hypoxia and distribution of HIF proteins in various hypoxic zones in tumor environment, (ii) tight regulation between hypoxia and angiogenesis, (iii) the role for hypoxia associated factors in maintenance of stem-like phenotype and development of CSCs, (iv) the role of hypoxia in cancer progression, metastasis, immunosuppression, and treatment resistance, (v) therapeutic strategies for re-engineer the hypoxic tumor microenvironment, (vi) therapeutic strategies considering the roles of CSCs in tumor development, metastasis and recurrence.

## Transcription factors of signaling pathways in hypoxia

The effects of hypoxia on tumor cells are mediated by the hypoxia inducible factor (HIF) family in major part [18]. The HIFs promote the expression of more than 150 genes, whose products coordinate the adaptive responses [19]. HIF-driven transcription encodes: vascular endothelial growth factor (VEGF), erythropoietin, transferrin and transferrin receptors, the enzymes required for glycolysis, anti-apoptotic factors, multiple growth factors [such as platelet-derived growth factor-B (PDGF-B), transforming growth factor beta (TGF-β), insulin-like growth factor-2 (IGF-2), epidermal growth factor (EGF)], and other proteins involved in normal homeostasis [20, 21]. Although, these factors are a part of adaptive response that allows them to compensate for reduced oxygen tension (and nutrients); hypoxic cancer cells overexpress them to increase survival, aberrant angiogenesis, extreme cell growth, and metastasis. The members of this family of transcription factors; HIF1, HIF2 and HIF3, are heterodimers that comprise α and β subunits. HIFα is a cytoplasmic protein regulated by oxygen levels, whereas HIFβ, a nuclear protein that is constitutively expressed, independent of hypoxic conditions [22]. HIF1α and HIF2α (also called EPAS-1/HRF/HLF/MOP2) in complex with HIF-1β (also known as ARNT) mediate the vast majority of HIF transcriptional activity. When oxygen supply is sufficient, HIF1α subunit is hydroxylated at proline residues through oxygen-dependent enzyme activity. By Hydroxylation of prolyl sites binding to von Hippel Lindau tumor suppressor (pVHL), HIF-1α undergoes degradation via the ubiquitin-proteasome system [23, 24]. Under hypoxic conditions, non-hydroxylated HIF-1α subunits translocated to the nucleus, where they combine with HIF-1β subunits to form heterodimers. The resultant heterodimer is the active HIF-1 factor binding to the hypoxia response elements of target genes that eventually drive the transcriptional responses [25]. HIF-1α appears to be expressed in most cell types, while the HIF-2α is mostly expressed in the endothelial cells (ECs) of the embryonic vasculature, neural crest-derived sympathetic ganglia, and of the developing pulmonary epithelial cells, kidney mesangial cells, and especially in renal cell carcinomas, in which pVHL, an E3 ligase for HIFα, is mutated. These two distinct, but highly related HIFs, have partially non-overlapping function and targeting a distinct subset of hypoxia-induced genes. An important distinction between HIF1α and HIF2α, is the specific stabilization pattern under different oxygen concentrations. Holquimvist-Mengelbier et al. [26], showed that HIF-2α, as opposed to the low HIF-1α activity, was strongly expressed and active at 5% oxygen (intermediate hypoxia), corresponding to well-vascularized tumor areas. Whereas, HIF-1α is predominantly active in 1% oxygen (severe hypoxia), under prolonged or chronic hypoxic conditions, the stabilization of HIF-1α is transient in this condition and HIF-2α is continuously accumulated in prolonged, severe hypoxia (1% O_2_).

### Tumor hypoxia and HIF protein expression

Hypoxic zones arise as a consequence of an imbalance between oxygen supply and consumption in solid malignant tumor, in which the oxygen pressure often reduces to a median of 0–20 mmHg (1%-2% or below). Whereas in normal healthy tissues oxygen tension is approximately 40 mmHg (~ 5%) in the liver and approximately 100 mm Hg (~ 13%) in arterial blood [4, 27]. Several mechanisms can contribute to the development of hypoxia in the tumor microenvironment. Perfusion-restricted hypoxia (also called acute, intermittent, transient, perfusion-limited or cyclic hypoxia), that can be transient, with insufficient delivery of oxygen due to aberrant blood vessels undergoing repeated cycles of closing and reopening, and consequential sluggish blood flow and fluctuations in oxygen supply. These constant fluctuations lead to cyclic periods of hypoxia and re-oxygenation that can lead to the development of a heterogeneous cell population within tumor [20, 28]. Diffusion-restricted hypoxia (also called chronic or permanent hypoxia) is the other type of tumor hypoxia and refers to the sustained restriction in oxygen diffusion by abnormal vascular network. Chronic hypoxia occurs when the tumor cells expand beyond 70 µm from the pre-existing nutritive blood vessels, which prevents an adequate delivery of oxygen [29]. At a farther distance from the blood supply (> 180 µm), the tumor cells can even become necrotic [30, 31]. Occasionally, anemic hypoxia can arise following a reduction in oxygen transport capacity by the blood. Anemic hypoxia can be either related to the presence of tumor or therapy-induced. Tumor tissue cannot fully compensate for the reduced oxygen levels, so it is more susceptible to anemic hypoxia [32].

Acute and chronic hypoxia are mainly mediated by HIF1α and HIF2α, respectively, and have often been reported to be associated with tumor progression and aggressive phenotype [26, 33]. However, as opposed to acute hypoxia, chronic hypoxia has also been shown to contribute to regression [34, 35]. Moreover, several studies have demonstrated that acute hypoxia increases cell survival and autophagy, selecting for cancer cells with stem cell characteristics, enhancing stem-like cell marker expression and endow resistance to radiotherapy [36–39]. Chenet al, showed that acute hypoxia, but not chronic hypoxia, induces genetic, molecular, biochemical, and cellular alterations promoting tumor cells with greater survival, heterogeneity, plasticity, tumorigenic capacity and resistance to anoikis, and thus enhanced ability for metastasis. In this study, HIF-1α expression was lost during chronic hypoxia, when HIF-2α mediated hypoxic responses may still be present [40].

## Hypoxia and angiogenesis

Tumor cells adapt to reduced oxygen levels by promoting the development of new blood vessels, a process termed angiogenesis [41]. Pro-angiogenic factors and their receptors including vascular endothelial growth factor (VEGF),VEGF receptor-1,-2 (VEGFR-1,-2), basic fibroblast growth factor (bFGF), platelet derived growth factor B (PDGF), insulin-like growth factor II (IGF2), adrenomedullin, and epidermal growth factor (EGF) are targets of the HIF transcription factors, and provide a molecular mechanism by which hypoxia regulates angiogenesis [42]. Several of these angiogenesis-related gene products, including iNOS, endothelin, adrenomedullin, and heme oxygenase 1, are also implicated in the modulation of local blood flow by regulating the vascular tone [43].

New vessels develop under hypoxic conditions but display substantial abnormalities, including; (i) an unusually elongated and tortuous shape, sometimes with blind-ends causing geometric resistance, and which leads to a disruption in blood flow and intermittent stasis, (ii) increased dilations and permeability due to insufficient smooth muscle cell layer, discontinuous endothelium or absent endothelial cell lining and basement membranes, (iii) a deficiency in pharmacological/physiological receptors and contractile wall components [20, 44].

Therefore, the tissue responses to ameliorate an impaired oxygen supply still fail [45]. Furthermore, due to a more access of metastatic cells to blood vessels, angiogenesis associated with a more invasive tumor phenotype [46, 47]. Angiogenesis is also an important step in carcinogenesis and transition from hyperplasia towards neoplasia [48].

### Signaling pathway for angiogenesis

Angiogenesis ismediated by the release of VEGF, the major hypoxia-inducible angiogenic stimulator, and this is driven by HIF.HIF-1 influences tumor blood flow through more complex mechanisms; HIF-1 targets different genes playing the role in vessel tone.

Information regarding the role of HIF2-α in hypoxia-related angiogenesis in tumor microenvironment is more controversial than for HIF-1α. Deletion of the HIF-1α gene in endothelial cells (EC), has been reported to reduce neovascularization and tumor growth, by disrupting the VEGF-mediated autocrine loop in EC, which is an essential component of solid tumor angiogenesis [49]. But, Skuli et al. [50], have demonstrated that HIF-2α gene deletion increased angiogenesis, albeit with more disorganized vessels resulting in poor perfusion and more hypoxic tumors in limb ischemia and autochthonous skin tumor models. They suggested that HIF-1α and HIF-2α act independently in response to local hypoxic stress, as HIF-1α promotes growth, proliferation, and morphogenesis in endothelial cells, while, HIF-2α is required for effective vessel remodeling and induction of a mature, functional vascular network. Conversely, this group of investigators had been previously reported that HIF-2α deletion resulted in reduced tumor angiogenesis, as well as increased vessel permeability and loss of integrity with a variable morphology in different adult organs [51]. These inconsistencies may be partly related to different expression patterns of HIF-2α in different tissues and its related cell type-specific signaling pathways [52]. HIF-1α and HIF-2α partially compensate for each other, however, they independently promote the modulations in the context of pathophysiological hypoxia to produce a functional vasculature [53, 54].

## The effect of hypoxia on cancer stem cells

There is mounting evidence that hypoxia affects the maintenance and functions of CSCs. CSCs constitute an undifferentiated stem-like cell subpopulation within the tumor heterogeneous cell types, which contributes to cancer initiation, progression, metastasis, therapeutic resistance and cancer relapse.

In solid tumors and hematological cancers, CSCs possess common properties to their normal stem cell counterparts, including self-renewal and the capacity to give rise to various cell types [55]. Furthermore, CSCs have the capacity for sphere formation. Although there are some potential markers suggested for CSCs in hematological cancers, definitive universal markers for CSCs in solid tumors remain unknown [56, 57]. CSCs appear to ‘educate’ neighboring cells to provide nutrients, cooperate in their evasion from the immune system, and create a microenvironment favoring tumor growth. CSCs can be differentiated into various cells that may be found in the tumor, which is associated with their high plasticity [58], establishment of a quiescent state with basal activity [59], enhanced survival ability in a stressful tumor environment with reduced oxygen and nutrient levels, and increased resistance to chemo-, radio- and immuno-therapy, and therefore utilized by tumors to escape from treatment and leads to recurrence [60]. Normal stem cells, such as hematopoietic stem cells, are retained in an hypoxic zone distant from the vasculature, which aids the maintenance of their stem cell properties [61]. While increased oxygen levels lead to the loss of these properties (stem and progenitor features referred to as stemness) [62]. Likewise, CSCs tend to localize to hypoxic regions within tumors, which probably favor the preservation the stem-ness, as well as to generate highly invasive and tumorigenic cells [63]. In conformity with this, Jogi et al., have reported that hypoxic cells in neuroblastoma (NB) adopt an immature phenotype [64]. It seems that cells in hypoxic areas, within the TME, are less mature and more invasive than oxygenated cells, and display a stem cell-like phenotype. [65].

HIF signaling plays a significant role in the modulation of various signaling pathways, which are exploited by CSCs to regulate stemness during hypoxic and therapeutic stress [66–68]. These pathways include Notch, Hedgehog, Hippo, Wnt (wingless)/β-catenin, Janus-activated kinase/signal transducer, activator of transcription (JAK/STAT), phosphatidylinositol 3-kinase/phosphatase, tensin homolog (PI3K/PTEN), and nuclear factor-kB (NF-kB) pathways. [69–71].

There is a growing body of evidence that implicates the reliance of CSCs on HIFs for the maintenance of their phenotype and function [68]. HIFs induce numerous gene products, including pluripotency related transcription factors, epithelial to mesenchymal transition (EMT) programmer, glycolysis-associated molecules, drug resistance-associated molecules, miRNAs and VEGF [68, 72].

### Hypoxia-mediated upregulation of pluripotency gene

HIF signaling enhances the maintenance of a CSC phenotype through the regulation of octamer-binding transcription factor 4 (Oct4), sex determining region Y box 2 (SOX2), kruppel-like factor 4 (KLF4), myelocytomatosis oncogene product (Myc), Tir nan Og (NANOG) and polycomb complex protein BMI-1 (BMI1) [72, 68].

Though both HIFs participate in CSCs survival and stemness maintenance [73, 74], hypoxia mediated transcription of stem-ness genes is differentially regulated by HIF-1α and HIF-2α. HIF-1α triggers Nanog, whilst HIF-2α activates Oct4 and c-Myc transcription [75–77]. Moreover, the HIFs contribute to the induction of CSC trait in different manner, with greater involvement of HIF-1α for survival functions and more specificity of HIF-2α for stem-ness properties (i.e. self-renewal) [78]. Bae et al. showed that the role of HIFs in CSCs function is dependent on the duration of exposure to hypoxia. HIF-1α enhances the expression of stem cell marker SOX2 and acute hypoxia-mediated cell invasion, while HIF-2α elevates the chronic hypoxia-mediated SOX2 and sphere formation in prostate cancer [79]. There is some evidence for the stronger induction of CSCs by HIF-1α in hypoxic areas during tumor development, and for HIF-2α as the main HIF factor under normoxic conditions that follow after cancer therapy, leading to a reduction in tumor size [80]. Consistent with this, Johansson and colleagues show that the expression of HIF-2α is significantly enhanced under normoxic and hypoxic conditions in glioma stem cells (GSCs). Also, the intracellular domain of CD44, a stem cell marker, is released in hypoxia, and binds to HIF-2α, but not HIF-1α, and subsequently upregulates hypoxia-induced stemness genes in GSCs [81].

### Hypoxia-mediated induction of EMT

There are experimental and clinical data suggesting that CSCs are strongly linked to EMT. EMT contributes to tumor aggressiveness, by promoting tumor cell invasion and migration, induction of stem cell phenotype, and subsequent therapeutic resistance leading to tumor recurrence [82–85]. Hypoxia, via HIF signaling, has been recognized to induce EMT and CSC features. During this phenomenon, tumor cells detach and acquire a mesenchymal phenotype, display the stemness properties including loss of differentiation, tumorigenesis and increased resistance to therapy [85]. Hypoxia/HIF-induced EMT and CSC phenotype mediated by similar signaling pathways consisting of NF-κB, PI3K/Akt, Wnt/β-catenin, Hedgehog and Notch [86]. A recent study also demonstrated that common genes are involved in EMT and a stemness phenotype, which contribute to tumor plasticity in response to anti-cancer therapies [87].

### Hypoxia-mediated miRNAs

Recent findings have been provided evidence that support the responsiveness of a variety of miRNAs to hypoxia that play pivotal role in many aspects of tumor development and malignancy, including angiogenesis, metabolic adaptation, EMT and CSCs sustenance. Hypoxia can down regulate the expression of miR-20, miR-22, miR-101 and let-7, as well as up regulate the expression of miR-21, miR-107, miR-181b, miR-210, and miR-373 in tumor cells. Hypoxia often up regulates oncogenic miRNAs and down regulates anti-tumorigenic miRNAs and thereby are associated with a poor clinical prognosis [86, 88]. However, it has been reported to be associated with an increase in anti-tumorigenic miRNAs such as miR-107. Therefore, more investigation is required to demonstrate the role of these miRNAs in stemness regulation and tumor progression under hypoxic conditions. Hypoxia-mediated miRNAs in TME that possess the ability of stemness regulation is summarized in Table 1.

**Table 1:** Dysregulation of the hypoxia-associated miRNAs modulates tumor angiogenesis, EMT and CSC features

miRNAs	Properties	Related cancers	Altered expression of miRNAs under hypoxic condition	Recognized mechanisms
miR-20 (a,b)	*Tumor suppressive* Anti-angiogenic, anti-proliferative, anti-invasive [89, 90] *Pro-oncogenic* Stemness preserving [91, 92]	Breast cancer [89] Osteosarcoma [90] Gastric cancer [91, 92] Colon cancer, pancreas cancer, prostate cancer [93]	Down-regulation	(miR-20a,b) regulation of VEGF [94] (miR-20b) Targeting HIF1α and STAT3, and inhibition of VEGF expression and modulate tumor angiogenesis [89, 90] (miR-20a) Targeting E2F1 and HIPK1, activation of Wnt-β-catenin signaling and sustain self-renewal ability of CSCs [91, 92]
miR-21	*Pro-oncogenic* Anti-apoptotic, pro-angiogenic, proliferative, invasive, chemoresistant, stemness preserving [95, 96]	Breast cancer [97, 98] Pancreatic cancer [96, 99] Prostate cancer, lung cancer [93] Head and neck cancer [100] Colorectal cancer [101–103] Gastric cancer [104, 105]	Up-regulation	Decrease of the tumor suppressor PTEN[106] and PDCD4[103] Increase expression of VEGF and HIF-1α and tumor angiogenesis [107] Increase CSC self-renewal capacity [108] and stemness properties [109, 110]
miR-22	*Tumor suppressive* Anti-angiogenic, anti-invasive, anti-proliferative, chemosensitive, radiosensitivite, pro-apoptotic [111–114] *Pro-oncogenic* Metastatic, stemness, preserving [115, 116]	Colon cancer [111] Hepatocellular carcinoma [112] Ovarian cancer [113] Cervical cancer [114] Breast cancer [115]	Down-regulation	Up-regulation of the tumor suppressor PTEN Suppression Of P21 And Induction Of P53-Dependent apoptosis [111, 117] Inhibition of c-Myc binding protein, reduction of human telomerase reverse transcriptase, and increased radiosensitivity [114] Inhibition of tumor suppressor *TET2*, increase of *Aim2, Hal, Igbt2*, and *Sp140*, and increase EMT and CSC self-renewal and stemness [115]
miR-101	*Tumor suppressive* Anti-invasive, stemness inhibitory [118]	Pancreatic cancer [119] Prostate cancer [120] Non-small cell lung cancer[121]	Down-regulation	Inactivation of epigenetic regulator EZH2, and inhibition of cancer CSC maintenance and EMT characteristics [119, 122]
miR-107	*Tumor suppressive* Anti-angiogenic, chemoresistant, stemness inhibitory [123, 124]	Breast cancer Colon cancer [125] Pancreatic cancer [93] Head and neck cancer [124]	Up-Regulation	Regulation of HIF-1β signaling, decrease of VEGF, and inhibition of VEGF mediated angiogenesis [125] Regulation of protein kinase Cε and stemness [124, 100]
miR-181b	*Pro-oncogenic *[126, 127]. *Tumor suppressive* Stemness inhibitory [128, 129]	Acute myeloid leukemia [126] Hepatocellular carcinoma [127] Glioblastoma [129] Non-small cell lung cancer [128]	Up-regulation	Inhibition of MLK2 and proliferation of cancer cells [126] Suppression of TIMP3, enhancement of MMP2 and MMP9 activity, and tumor progression and invasion [127] Down regulation of Notch2 in CSCs, decrease stem markers, Suppression of tumorsphere formation, and increases Chemosensitivity related to CSCs [128]
miR-200 (a,b,c)	*Tumor suppressive* Anti-invasive, stemness inhibitory [118, 130]	Gastric cancer [131] Breast cancer [132] Prostate cancer, pancreatic cancer, colon cancer [118] Nasopharyngeal carcinoma [130]	Down-regulation	Targeting the ZEB1/ZEB2, suppression of Wnt/β-catenin pathway, increase E-cadherin, and inhibition of EMT[131] and CSC phenotype [132, 133] Down-regulation of Bmil-1, Suz12, and Notch-1, regulating the CSC and EMT phenotypes and functions and inhibition of CSC formation [118] Targeting the MYH10, and inhibition of migration and invasion [130]
miR-210	*Pro-oncogenic* Pro-angiogenic, proliferative, invasive, metastatic, stemness preserving [134]	Breast cancer [134, 135] Colon ccancer [136]	Up-regulation	Increase of VEGF and CAIX expression, and tumor angiogenesis Targeting of E-cadherin mRNA, up-regulation of E-cadherin transcription repressor (Snail) and suppression of E-cadherin expression, increase of CSC metastasis, proliferation, and self-renewal capacity [134] Suppression of RAD52, reduces DNA repair and results in hypoxia-related genetic instability in cancer [137]
miR-373	*Pro-oncogenic* Proliferative, invasive, metastatic, stemness preserving [138, 139]	Breast cancer [138] Colorectal cancer [139]	Up-regulation	Transactivation of E-cadherin and metastatic develpment [140, 141] Activation of Nanog and Hedgehog signaling pathways, and enhancement of cancer cell self-renewal capacity and stemness [139] Suppression of RAD23B and RAD52, reduces DNA repair and results in hypoxia-related genetic instability in cancer [137]
let-7 (a, b, c, d, e, f, g)	*Tumor suppressive* Anti-invasive,stemness inhibitory [142–144].	Breast cancer [145] Head and neck cancer [100] Oral cancer [142] Prostate cancer [143] Pancreatic cancer [144]	Down-regulation	Regulation of PTEN, CSC marker Lin28b, suppression of EMT and CSC phenotype [142–144] Blockage of Wnt signaling and suppressing self-renewal and stemness of CSCs [145] Inhibition of EZH2, and suppression of CSC characteristics [146]
miR-26a	*Tumor suppressive* Anti-invasive, stemness inhibitory [147–149]	Gastric cancer [150] Hepatocellular carcinoma [149] Breast cancer, prostate cancer, pancreatic cancer, nasopharyngeal carcinoma [118]	?	Suppression of HOXC9 and inhibits its metastatic and stemness features of self-renewal [150] Inhibition of EZH2, and suppression of CSC characteristics [147, 148] and EMT [149]

### Hypoxia-mediated angiogenic factors

HIFs up regulate the expression of angiogenic factors, particularly VEGF in CSCs and promote tumor angiogenesis [151, 152]. In addition to stimulation of angiogenesis, hypoxia-induced VEGF is known to drive EMT and maintain CSC stemness, and by these mechanisms contributes to tumor invasion and metastasis [153, 154].

### Hypoxia-mediated metabolic adaptation

Hypoxia-induced metabolic reprogramming can also contribute to CSC maintenance and resistance [68]. In most cases, the principal metabolic pattern in normal stem cells is glycolysis whereas differentiated cells primarily perform oxidative phosphorylation (OXPHOS). Meanwhile, CSCs do not resemble the metabolic pattern of normal stem cells. Indeed, both glycolysis and OXPHOS are metabolic engines in CSCs, and predominant metabolic pattern depends on the type of tumor and TME stimuli [155]. The hallmark of hypoxia-induced metabolic reprogramming is a shift in ATP production from OXPHOS to glycolysis. Under hypoxic conditions, HIF-1α (and HIF-2α) that is stabilized in CSCs, triggers the expression of genes involved in metabolic adaptation (e.g., GLUT1, LDH -A, PDK) [77]. Consequently, lactate increased and levels of ROS are reduced, in turn leading to CSC protection and potentially therapy failure [156]. Evidences show that high level of lactate and acidic stress in the microenvironment induce CSC phenotype [157], tumor invasiveness, and impair the immune response [158, 159]. Acidic stress is considered to be a mechanism for HIF-2α induction, in which HIF-1α can upregulate HIF-2α activation via metabolic pathways [160].

There is some evidence for a link between high levels of aldehyde dehydrogenase (ALDH), the enzyme responsible for oxidizing intracellular aldehydes, and response of CSCs to hypoxia. Shiraishi and colleagues, showed that under hypoxic conditions, the expression of ALDH1A1 is associated with the overexpression of HIF-1α, but not HIF-2α, and stemness in breast cancer stem cells (BCSCs) [161]. Kim and colleagues, however, showed that ALDH was highly associated with the HIF-2α expression of breast cancer cell lines in vitro and self-renewal ability of BCSCs in mouse model of breast cancer [162]. Regardless of HIFs, overexpression of the membrane-bound ectoenzymes carbonic anhydrases (CAIX and CAXII) and monocarboxylate transporter-4 (MCT-4) in response to hypoxia, promotes a CSC phenotype and function, by modifying acidic pH of hypoxic cancer cells with a high rate of glycolytic metabolism [163, 164].

### Hypoxia-mediated quiescence

By modulation of the process of metabolic adaptation, apoptotic pathways, cell cycle and self-renewal, hypoxia, maintains the CSCs in the quiescent state. Quiescence defined as a protective response to adverse condition, enabling cells to conserve the proliferative potency and eliminate DNA damage [165]. Non-dividing quiescent CSCs that can survive after conventional therapy targeting rapidly dividing cells, are responsible for the failure of cancer therapy and tumor relapse [166]. Prati and colleagues demonstrated that under chronic hypoxic stress, breast cancer cells enter into quiescent state, characterized by cell-cycle arrest in G0/G1 and lower levels of metabolism. These dormant cells have a CSC phenotype and sphere-forming capacity [167].

One of the major regulators that contributes to the induction of cell-cycle arrest, and quiescence, during hypoxia, is HIF-1α, that acts independently of p53 and causes the up-regulation of cyclin-dependent kinase inhibitors p21 and p27, hypophosphorylation of RB and suppression of cell cycle gene CDC25A [168, 169]. However, it has been reported that the de-depression of cyclin-dependent kinase inhibitors, such as p21, is not essential for HIF-1α-induced quiescence [170]. Also, HIF-1α^+^ quiescent stem-like cells are associated with increased sphere-forming capacity and higher tumorigenicity in glioblastoma patients [171]. Although, HIF-1α can promote cell-cycle arrest by inhibiting c-Myc activity [172], HIF-2α has been shown to stimulate the progression of the cell-cycle and neoplastic growth of cancer cell, via functional enhancement of c-Myc [76]. The induction of quiescence by HIF-1α may appear to contradict some evidence from its role in cancer growth. However, HIF-1α may temporally maintain cancer cells in a quiescent state and conserve their survival as long as oxygen supply is available, where they become more invasive.

### Hypoxia-mediated resistance

Hypoxic CSCs also have a high expression of a number of Adenosine triphosphate–binding cassette (ABC) transporters, including multidrug resistance gene 1 (MDR1 or ABCB1) and its product permeability glycoprotein (P-glycoprotein), multidrug resistance-associated protein (MRP1), breast cancer resistance protein (BCRP or ABCG2 or MXR) and ABCB5 [78]. These membrane transporters as a feature of stem-like cells, and contribute to transporting cytotoxic materials out of cell. CSCs exploit the BCRP to offset the detrimental effect of heme or porphyrin accumulation, under hypoxic condition [173]. The ABC transporters, further, provide a high capacity efflux system, leading to chemotherapy failure and highly drug-resistant tumor formation [174]. HIFs play the role in the activation of drug transporter in CSCs upon hypoxic condition. The HIF-mediated increased expression of these transporter reduce the effect of chemotherapeutic agents, including paclitaxel, gemcitabine and Imantinib [175, 176]. Both HIF-1α and HIF-2α participate in the up-regulation of ABC transporters and chemo-resistance, but in different capacities. With a central role of HIF-1α for MDR1 activation and partial participation in BCRP and MRP1 activity, as well as more responsibility of HIF-2α for BCRP activation and less relevance to MDR1 control [177].

## The role of hypoxia in cancer progression, metastasis, immunosuppression, and treatment resistance

Hypoxia can substantially affect the malignant progression and therapeutic response of solid tumors by change the transcriptome and regulating the production of proteins in tumor cells that modulate immune privilege and limit anticancer immunity [178]. Thus, hypoxic condition often linked to a poor prognosis of many cancers. Conversely, some evidences implicate the augmentation of anti-tumor response by hypoxia associated factors.

### Hypoxia-induced cancer progression and metastasis

**Fig. 1:**
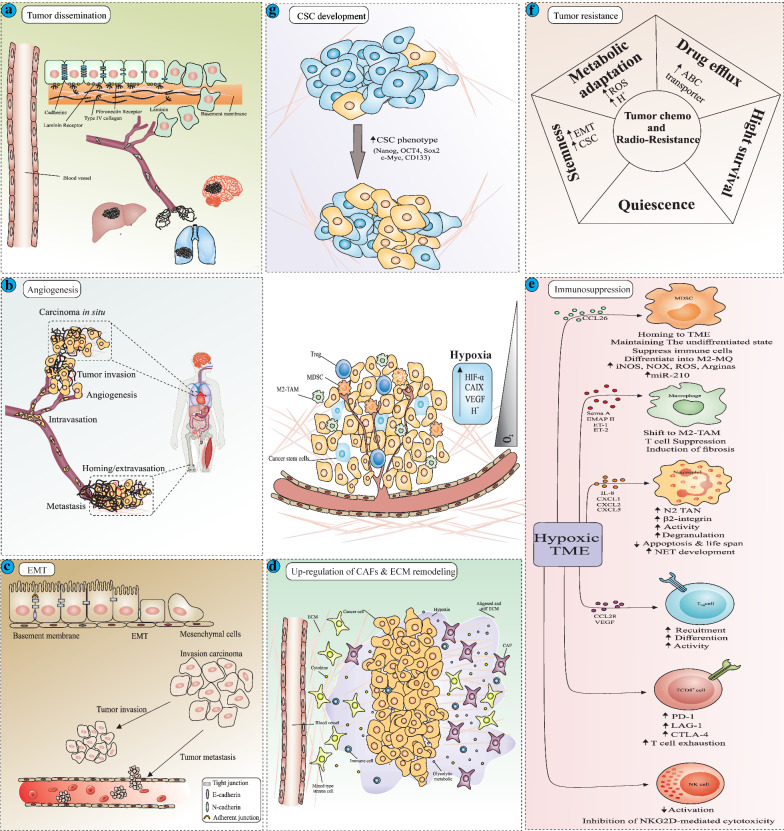
The diagram displays the responses to reduced oxygenation within the tumor microenvironment. Hypoxia promotes tumor invasion, metastasis and resistance through several ways. **a** Hypoxia induces detachment of tumor cells by weakening the connections between cells and their extracellular matrix supporting them, and promotes dissemination of tumor cells to the various organs of the body. Thereby hypoxia triggers the metastatic spread of tumor. **b** Hypoxia stimulates angiogenesis, and provides more opportunity for detached tumor cell to inter into the circulation and migrate via the newly formed vessels. Thereby hypoxia enhances invasive and metastatic spread of solid tumors to another region. **c** Hypoxia induces EMT, in which tumor cells detach, lose the epithelial feature, acquire a mesenchymal phenotype and display the stemness properties including loss of differentiation, tumorigenesis and aggressiveness. EMT extensively contributes to promoting of tumor cell invasion and migration. **d** Hypoxia up-regulates CAFs that produce the excessive altered ECM, which supports tumor growth and metastasis. **e** Tumor hypoxia promotes secretion of cytokines and chemokines that recruit pro-tumor immune cell and suppress anti-tumor response of various types of immune cells. **f** In response to hypoxia, tumor cells exploit a number of mechanisms including, extrusion of cytotoxic drug by ABC-transporters, exhibiting quiescent state, acquiring metabolic adaptations and displaying stemness features, which can contribute in chemo-, radio- therapy failure. **g** Hypoxia acts as a niche condition, to accumulate the CSCs enhancing tumorigenesis and resistance. *EMT* epithelial to mesenchymal transition, *CAF* cancer-associated fibroblast, *ECM* extracellular matrix, *MDSCs* myeloid-derived suppressor cells, *TAM* tumor-associated macrophage, *TAN* tumor-associated neutrophil, *Treg* regulatory T lymphocyte, *NK cell* natural killer cell, *CSC* cancer stem cell

Hypoxia may facilitate the dissemination of cells from the tumor to the other region of the body, by reducing the strength of interactions between cells and their supporting extracellular matrix (Fig. 1a). Czekayet al. [179], have shown that plasminogen activator inhibitor-1 (PAI-1) has a de-adhesive activity, as well as other de-adhesion molecules that include: thrombospondins (TSPs) 1 and 2, tenascin-C, and SPARC(secreted protein, acidic and rich in cysteine) [180]. These proteins can reverse the cell adhesion process and disrupt the integrin-mediated cell attachment. Consistent with this, high levels of PAI-1 have been reported to be associated with a poor prognosis in metastatic human cancers [181, 182].

Furthermore, vascularization, as an important consequences of HIF signaling, has emerged as a fundamental feature of neoplastic growth, tumor progression and distant metastasis. Excessive blood vessel formation gives the weakly attached tumor cells a greater chance to enter into the circulation and migrate (Fig. 1b) [46, 47]. Thereby, hypoxia triggers the escape of tumor cells from the hostile environment and enhances the invasiveness and metastatic spread [183, 184].

EMT is another process promoted by hypoxia. Tumor cells, which undergo EMT, are reprogramed to have greater mesenchymal features, reduced intercellular attachments and cell-to-cell contacts, increased motility, and conversion to a stem-like phenotype. Thereby, hypoxia-induced EMT increases the aggressiveness and metastatic potential of these cells (Fig. 1c) [84, 85]. Expression of lysyl oxidase (LOX) and lysyl oxidase-like 2 (LOXL2) and HIF-1 accumulation, downregulates E-cadherin and leads to EMT, which contributes to renal, breast and cervical cancer progression and metastasis [185–188]. HIF-1, also contributes to EMT induction in VHL-null renal cell carcinoma by indirect repression of E-cadherin, mediated by the expression of TCF3, ZFHX1A, and ZFHX1B [189]. Moreover, HIF1-α increases the transcriptional repressor SNA, and downregulates E-cadherin in ovarian carcinoma cells [190]. Kang and colleagues, also showed that the hypoxia-induced EMT is associated with increased CXCR4 expression via of aberrant demethylation of its promoter in lung cancer cell, in which, CXCR4 siRNA inhibits the hypoxia-induced EMT and acquisition of stemness [191].

Moreover, hypoxia has been shown to influence fibroblast reprogramming and upregulate cancer-associated fibroblasts (CAFs) in HIF-1α dependent manner with a metabolic shift towards glycolysis, and that leads to an increase in lactate production. This catabolite is produced by highly glycolytic CAFs and can be used by cancer cells and enhances cancer proliferation, which describes a negative outcome of HIF-1 accumulation in fibroblasts [192, 193]. CAFs share several characteristics with normal fibroblasts that are activated, but they possess oncogenic functions substantially due to production of altered ECM, which supports tumor growth and dissemination (Fig. 1d) [194]. The ECM produced by cancer cells and CAFs in hypoxia, differs from the normoxic ECM and continuously undergo remodeling, which facilitates angiogenesis [195], tumor cell migration and metastatic spread [196, 197].

### Hypoxia-induced cancer immunosuppression

By influencing various types of immune cells, hypoxia has been associated with immunosuppression and subsequent tumor progression (Fig. 1e) [198].

The characteristics of the hypoxic tumor microenvironment, are frequently reported to correlate with the accumulation and augmentation of MDSCs. Hypoxic tumor cells can produce CCL26 to attract MDSCs and their homing to the primary tumor [199]. The major feature of these heterogeneous undifferentiated cells, is the exhaustion and suppression of immune cells, which in tumor context leads to evasion from immune surveillance and bypass the blockade of immune checkpoints. One of the underlying mechanism reported in 2017, in which hypoxia/HIF-1α induces ectonucleoside triphosphate diphosphohydrolase 2 (ENTPD2/CD39L1) in cancer cells result in overexpression of extracellular 5′-AMP, which maintains MDSC in undifferentiated and immunosuppressive state in the tumor stroma [200]. However, Corzo et al. [201] reported that HIF-1α mediates, the rapidly differentiation of MDSCs into macrophages with immune-suppressive features upon their arrival at tumor sites. They also described differences between MDSCs in tumor site, contrasted with MDSCs located in peripheral lymphoid organs, that acquire the ability to suppress antigen-nonspecific T cell, as a result of hypoxia induced upregulation of inducible nitric oxide synthase (iNOS) and arginase I, and associated downregulation of both nicotinamide adenine dinucleotide phosphate–oxidase 2(NOX2) and reactive oxygen species (ROS). In addition, hypoxia can enhance MDSCs activities through a HIF1α-dependent mechanism and subsequently elevates miR-210 expression. miR-210 regulates the function of MDSCs by increasing arginase activity and nitric oxide production [202]. Although it is widely accepted that MDSCs have immunosuppressive activity, some studies have found immunostimulatory properties of M1 type MDSCs, compared to the suppressive M2 phenotype [203]. SIRT1 induction and mTOR/ HIF-1α-dependent glycolytic reprogramming, has been found as an underlying mechanism in lineage differentiation of MDSCs into the tumor-suppressing M1 phenotype [204].

Under hypoxic condition, tumor cells promote the secretion of chemoattractive substances, including hypoxia-induced Semaphorin 3A (Sema3A), Endothelial-monocyte-activating polypeptide II (EMAPII), ET-1 and ET-2, which promote recruitment of macrophages [205–207]. As hypoxia triggers the switch to glycolysis and increases lactate and H^+^ production, it can also promote M2 polarization of tumor-associated macrophages [208]. Activated M2 macrophages, in contrast to their classically activate M1 counterparts, have more capacity to induce angiogenesis and tumor progression and metastasis. Besides accumulation of macrophages, hypoxia strongly augments macrophage-mediated T-cell suppression in a HIF-1α dependent manner. Nevertheless, both of HIF1-α and HIF2-α have been shown to be pivotal for macrophage infiltration and immune suppression [209, 210].

Neutrophils infiltrating into the tumor microenvironment, are known as tumor-associated neutrophils (TANs), and are a heterogeneous population including anti-(N1) and pro-tumor (N2) phenotypes [211]. Tumor hypoxia is one of the factors can influence neutrophil plasticity [212]. However, the mechanism by which hypoxia/HIFs affects the N1 and N2-polarization of TANs remains to be clarified. Reciprocally, neutrophils enhance micro-environmental hypoxia by depleting localized oxygen, resulting in upregulation of hypoxia-inducible genes [213]. Hypoxia, through HIF-dependent production of cytokines (IL-8) [214], and chemokines (CXCL1, CXCL2, and CXCL5) [215] can recruit TANs. In addition, hypoxia upregulates the expression of the cell surface adhesion molecule β_2_ integrin in neutrophils and enhances their function, in a HIF-1α dependent manner [216]. HIF-2α is a critical mediator of neutrophil recruitment to colon tumors and subsequent increase of colon carcinogenesis, by upregulating the potent neutrophil chemokine CXCL1 [217]. Local hypoxia have been shown to promote neutrophil extracellular traps (NETs) [218], which consist of expelled DNA and various proteins such as neutrophil elastase (NE). NETs promote angiogenesis, cancer progression and metastasis [219–221]. Moreover, TGF-β, as a known target of HIF-1, reported to induces a pro-tumor N2 phenotype of TANs [222]. It is appear that hypoxia causes a shift of N1 toward N2 TANs, which supports tumor progression and metastasis [212].

The metabolic competition between tumor cell and infiltrating lymphocytes, leads to a limitation of glycolysis in tumor-infiltrating T cells [223–225], hypoxia can suppress the anti-tumor activities of T cells through the other mechanisms mediated by HIF. Regulatory T-cell (Treg) repress the anti-tumor responses and addressed as an important limitation of cancer immunotherapy [226, 227]. Tumor cells under hypoxic conditions, upregulate the expression of chemokines and cytokines, such as CC-chemokine ligand 28 (CCL28) and VEGF, which attract Tregs (CD4 + CD25 + FoxP3 + nTreg) to suppress tumor-reactive T cells and promote angiogenesis [228–231]. Hypoxia induces FoxP3 and promotes the differentiation of Tregs (iTregs) from naive T cells through T-cell intrinsic HIF-1α pathway [232]. Indeed, hypoxia is considered to be an indicator of the inflamed microenvironment, and an increase the proportion of Tregs is an anti-inflammatory mechanisms to restrict the detrimental effects of inflammatory hypoxia [233]. In contrast, there is emerging evidence that shows that HIF-1α possesses a deleterious effect in suppressive activity and stability of Tregs by degradation of FoxP3, and implicates a role for HIF-1α in development and function of Tregs [234, 235].

Another mechanism by which hypoxia represses the anti-tumor response involves the modulation of immune checkpoints. HIF1-α, and occasionally HIF2-α, upregulates the immune inhibitory molecule programmed cell death ligand-1 (PD-L1; also termed B7-H1) in hypoxic-tumor cells, macrophages and MDSCs [236–239]. Furthermore, HIF-1α enhances the expression of inhibitory receptors including programmed cell death 1 (PDCD1; also termed PD-1), lymphocyte activating gene 3 (LAG3, also termed CD223) and CTLA-4 in CD8^+^ T cells [240, 241]. Hence, hypoxia promotes T cell exhaustion and tumor resistance to CTL-mediated lysis.

In contrast to what is described above, a potential adverse effect of hypoxia on tumor-reactive T cells, has been shown; in respect of the stimulatory role mediated by HIF1-α and HIF-2α in CTL proliferation and function [242]. Mohapatra et al. found that in glioblastoma (GBM) [243] there was a downregulation of immunosuppressive enzyme tryptophan-2,3-dioxygenase (TDO2) involved in tryptophan (Trp) catabolism, in a HIF1α-dependent manner, with increased T cell proliferation. While, T cell proliferation is inhibited by TDO2-expressing GBM cells under normoxia. Its assumed that tumor cells downregulate TDO2 to conserve Trp through HIF1-α in a nutrient-deficient hypoxic microenvironment. Consistent with this, Tyrakis et al. [244] were able to show an enhancement of proliferation, survival and anti-tumor capacity of CD8^+^ T cells by hypoxic induction of 2-hydroxyglutarate through the HIF-1α dependent fashion. Moreover, hypoxic CTLs exhibited higher intrinsic cytotoxic capacity and improved function in tumor regression compared to normoxic CTLs [245]. However, hypoxia was shown to negatively regulate the interplay between NK cells and tumor cells. In hypoxia HIF-1α upregulates the metalloproteinase ADAM10 that leads to shedding of the NK cell-activating ligand, MICA, from the surface of tumor cells, results in resistance to cytotoxic killing by NK cell. Mechanism of this NKG2DL shedding involves impaired nitric oxide (NO) signaling [246, 247]. Thereby hypoxia contributes to escape from innate immunity. In contrast, the supporting role of hypoxia in NK cell priming and activation, in synergism with IL-15, has been reported [248, 249].

### Hypoxia-induced cancer resistance

Resistance to conventional treatment is a major obstacle in clinical oncology that lead to cancer relapse. Oxygen deprivation may cause in resistance to ionizing radiation, multiple forms of chemotherapy and photodynamic therapy. Several mechanisms of hypoxia-induced resistance related to cellular adaptations to poor oxygenation and nutrition (Fig. 1f). Hypoxia promotes cell cycle arrest and a quiescence cellular state that reduces the susceptibility to external stress during radiation or chemotherapy, especially therapies targeted at rapidly dividing cells [63]. Hypoxia increases the synthesis of certain proteins in subclones, undergoing selection pressure. Consequently, during clonal selection and genome changes, tumorigenic subpopulations are formed which related to a lack of differentiation, defective senescence and apoptosis, augmented spreading and metastasis can lead to further resistance to therapy [29, 63, 250]. Moreover, hypoxia modulates mitochondrial activity [cell apoptosis and necroptosis, as well as generation of reactive oxygen species (ROS)] and induces mitophagy. Thereby, hypoxia reduces the susceptibility of tumor cell to drug- and radiation-induced apoptosis and ROS formation [251, 252]. Modulation of autophagy and p53 known as another mechanism involved in the hypoxia-induced resistance to chemotherapy, however their molecular pathways remained largely elusive [253, 254].

In addition, loss of oxygen required for the cytotoxic function of the certain chemotherapeutics, orientation of chaotic and malfunctioning blood vessels, and augmentation of metastasis [255], reduction of drug delivery and cellular uptake due to tissue acidosis, overexpression of the multidrug resistance (MDR1) gene [256] and its product P- glycoprotein (drug efflux pump) [257, 258], enhanced production of nucleophilic substances such as glutathione, which compete with the target DNA for alkylation, and upregulated enzymatic repair of DNA, are other possible mechanisms by which hypoxia reduces the responsiveness to chemotherapeutic agents [259].

Vasculogenic mimicry (VM) is a major factor in metastasizing breast cancer and resistance to anti-angiogenic drugs. Hypoxia, EMT and CSC are considered as important factors in the formation of VM. Melatonin is also a hormone derived from amino acids, with many anti-tumor effects. There are numerous antitumor effects of melatonin, but its effect on breast cancer VM formation has not yet been investigated. Therefore, we investigated the impact of melatonin in breast CSCs on VM formation via the EMT cycle under hypoxia conditions. We evaluated the impact of melatonin on the EMT markers expression. As a CSC marker in the MDA-MB-231 cell line, CD44 + CD24-phenotype was 80.8 percent whereas in the MCF-7 cell line it was 11.1 percent. The expression of HIF-1α in the VM-positive breast cancer cell line MDA-MB-231 was up-regulated and thus influenced the expression of the EMT markers E-cadherin, vimentin, snail and MMP9. Melatonin in breast CSCs had major effects on EMT and VM formations. Melatonin may hinder VM formation by influencing the key molecules taking part in VM structure and the EMT formation. In addition, our results demonstrate obviously that melatonin is a hormone with important anti-cancer activity. It may probably improve breast cancer management via overcome anti-angiogenic drug resistance [260].

Notwithstanding, these mechanisms that are principally mediated by the HIF-1, the roles of HIF-2 in resistant to anticancer therapy is recently reported, including its roles in affecting HIF-induced autophagy and hampering p53-mediated apoptosis [261, 262].

Recent evidence also suggests that resistance is largely linked to the function of CSCs [166]. As previously described, hypoxic areas within the TME provide a niche for development and accumulation of CSCs, which are poorly differentiated and more invasive, enhancing tumorigenesis and resistance to chemo-, radio- and immuno-therapy (Fig. 1g).

## Strategies to re-engineer the tumor microenvironment for treatment

Hypoxia is a major feature of solid tumors and an important impediment to conventional cancer therapy. Identification of the predominant cellular and molecular mechanisms in hypoxic TME presents the first step to design of effective anti-cancer strategies in the context of solid tumors.

### Targeting HIFs as a therapeutic strategy

Given that HIF proteins are the master regulators of oxygen homeostasis in hypoxia, targeting HIFs is an attractive strategy in the treatment of tumors. Several approaches for targeting hypoxia and HIF may be achieved by exploiting; (1) hypoxia-activated prodrugs (HAPs) or drug; (2) specific targeting of HIFs; (3) targeting of downstream HIF signaling pathways and important pathways in hypoxic cells (such as mTOR and UPR).

HAPs, which also known as bioreductive alkylating agents, can directly or indirectly regulate HIFs. HAPs are inactive compounds that can be activated to cytotoxic drugs in hypoxic cells or tissues, spontaneously or via one/two electron oxidoreductases [263]. Several HAPs, including PR-104, Evofosfamide (TH-302), and apaziquone (EO9), have undergone preclinical study and clinical development. However the result of clinical trials showed some disappointments and thus far there is no FDA approved HAP [264]. Fortunately, promising progress has been reported in phase II clinical trial on TH-302 in combination with gemcitabine for pancreatic cancer [265], and with adriamycin or doxorubicin for soft tissue sarcoma [266], and phase III studies are underway. Furthermore, TH-302 sensitizes prostate cancer to immunotherapy with checkpoint blockades CTLA-4 and PD-1. Combination therapy with this hypoxia-prodrug and checkpoint blockade drive a more influx of T cells into hypoxic zones and reduce MDSC and granulocytic subsets in tumor environment [267].

The other approaches for overcoming hypoxia, involve direct or indirect targeting of HIF and targeting of downstream HIF signaling pathways. The agent exploited to inhibit the HIF response to hypoxia include inhibitor of HIF-1α mRNA expression (such as antisense oligonucleotide EZN-2968), HIF-1α protein translation (including inhibitors of topoisomerase I/II, receptor tyrosine kinase, cyclin-dependent kinase, oncogenic pathways, thioredoxin reductase, activators of p53 and microtubule disrupting agents), HIF-1α protein degradation (including Hsp90 inhibitors such as 17-AAG and 17-DMAG), HIF-1α DNA binding (such as doxorubicin and daunorubicin) and HIF-1α transcriptional activity (Bortezomib (PS-341) and chetomin) [268]. These inhibitors have been described in detail in several comprehensive reviews [18, 268]. Here we will summarize the strategies targeting the main pathways involved in hypoxia in tumor environment.

### Targeting angiogenesis as a therapeutic strategy

One good example of targeting gene products at downstream HIF signaling, is anti-VEGF therapeutic strategy. Anti-angiogenic approaches including monoclonal antibodies (mAb) targeting VEGF or small-molecule inhibitors targeting VEGF receptors can obviate the deleterious effects of angiogenesis in TME favoring tumor growth [269].

Bevacizumab (avastin) is a humanized anti-VEGF IgG1 mAb that has been approved for numerous recurrent and metastatic cancer. Ramucirumab is the anti-VEGFR-2 IgG1 mAb and approved for the treatment of advanced and metastatic several types of cancer, including stomach, colorectal and non-small cell lung carcinoma (NSCLC) [269]. Another anti-angiogenic approach has been achieved through targeting Angiopoietin-1 or 2 (Ang-1,2), using Trebananib. Angiopoietin displays broad expression in the remodeling vasculature of tumors and binds to its receptor, Tie-2. Trebananib is a “peptibody” madeas an antibody and peptide-Fc fusion protein that impairs the interaction between Ang-1,2and Tie-2, leading to the suppression of angiogenesis and tumor growth [270, 271].

Over the last few years, several small-molecule kinase inhibitors have been developed for treatment of different cancers. For instance, sorafenib, regorafenib, lenvatinib, nintedanib, sunitinib, cabozantinib and pazopanib that can inhibit several protein kinases including VEGF receptor (VEGFR), approved by the FDA for treatment of different cancers and as adjuvant for cancer chemotherapy. In addition, 1,2-disubstituted benzimidazoles, the newly synthesized compounds exhibits promising cytotoxic and VEGFR-2 inhibitory [272].

Despite the reported clinical benefits, anti-angiogenic therapies have been not shown to be always effective. Hypoxia induced in response to reduced perfusion during anti-VEGF treatment promotes a greater dependence on hypoxia adaptations, in particular, mediated by HIF1 and HIF2, the unfolded protein response (UPR) and ATF4 [269, 273]. For example, anti-angiogenic therapy promotes metabolic changes through HIF-1 expression, in turn increases lactic acid and carbonic acids production. Therapy against VEGF, also, can promote the inherent selection of tumor cells, which adapt to more hypoxic conditions. Furthermore, exaggerated hypoxia resulted following anti-VEGF treatment which favored a sustained CSC phenotype, and which in turn may contribute to tumor maintenance and resistance to therapies [274]. Hence, an effective anti-angiogenic therapy may be achieved in combination with inhibitors of tumor hypoxic adaptation.

### Targeting hypoxia-induced metabolic changes as a therapeutic strategy

The accumulation of lactate in tumors results in reduced intracellular pH (pHi). Moreover, tumor cells in response to hypoxia, upregulate carbonic anhydrases and the production of CO_2_, which contributes to cellular acidification [275]. To counteract acidification, hypoxic tumor cells upregulate a number of membrane transporters, exchangers and pumps and release the lactic acid into extracellular space. Further, they upregulate extracellular carbonic anhydrases (CAs) catalyzing the hydration of CO2 to bicarbonate, which pumped in and in turn increases the pHi and acidifies the extracellular pH (pHe) [276]. The other important transporters, involved in the export of proton from cells, include the sodium hydrogen exchanger 1 (NHE1) and monocarboxylic acid transporters (MCTs) [277, 278]. Also, ion channels such as transporters, exchanger, pumps and voltage gated sodium channels, endow the tumor with the proliferative, invasive and metastatic ability [279]. These channels modulate tumor cell survival, proliferation, resistance to apoptosis, cell adhesion, motility and extracellular matrix invasion [280, 281]. Given that the hypoxic tumor is dependent on glycolysis and pHi-regulating systems, ion channels have been recently proposed as a potential target for selective therapeutics. The strategy of ion channel blockades accompanies maintaining the low intracellular pH in malignant cells, which induce acid-mediated metabolic collapse following by apoptosis or necrosis [282, 283]. For instance, targeting of NHE1 recently reported to be effective in the treatment of glioblastomas, as highly glycolytic and strongly pH-dependent malignancies [284, 285]. It has also been shown that proton pump inhibitors (PPI, V-ATPase blocker) increase the uptake and effect of cytotoxic drugs in chemo-resistant epithelial ovarian cancer [286]. Furthermore, MCTs (in particular, MCT-4, as hypoxia-inducible isoform and MCT-1) and CAs (in particular, CAIX and CAXII) have been offered the promising therapeutic targets for various cancer types, and recently, a large variety of inhibitors that target their different isoforms are being tested [277, 287]. Additionally, targeting of CAIX emerged as attractive strategy to eliminating CSCs in hypoxia [288, 289].

#### Targeting hypoxia-induced metabolic changes to improve the efficacy of immunotherapy

A relatively low pH is a hallmark of solid tumor environments, with pH 6.0–6.5, compared to pH 7.5 present in normal environments [290]. High acidity associated with solid tumors acts as a barrier for immune-based therapies such as checkpoint inhibitors or adoptive T-cell transfer [291]. This could, in part, be due to the adverse effect of acidity on different immune cells. Acidity leads to a reduced lifespan of CD8^+^ memory T cells [292], raise the activation threshold of T cells and modulate the activity of T cells by up-regulating the immune checkpoints such as CTLA-4 [293]. High extracellular lactate can induce a tolerogenic phenotype of dendritic cells, characterized by reduced IL-12 and increased IL-10 production in response to TLR stimulation, impaired migratory response to chemokines and defective metabolism [294]. Chronic long-term exposure to acidic milieu of tumor, also, impairs NK cell activation, function and survival [293]. On the other hand, tumor acidity recruits MDSCs [294]. There is also evidence suggesting that low a pH decreases T cell infiltration and their homing into the TME.

Recently, Thomas and coworkers [295], examine the effect of pH buffering and neutralizing tumor acidity by oral bicarbonate administration, on anti-tumor responses to checkpoint immunotherapyand observed the increase of anti-tumor immunity in multiple cancer modelsOf note, acidity-reversing drugs appear to be artificially raised the pH of tumor vasculature, allowing to increase T cell homing at this site. In addition, pH gradient created between intracellular and extracellular environments affects the drug absorption and metabolism, also suppresses cytoplasmic retention of cytotoxic anticancer agents [296], and thereby facilitates the acquired drug resistance.

### Strategies to limit hypoxia-induced radio- and chemo-resistance

Recent studies show that a combination of supplemental oxygen therapy with cytotoxic drug or radiation therapy enhanced their effectiveness [297]. Anti-cancer drugs mostly kill cells in an oxygen-dependent manner and require oxygen for maximal activity. Platinum based chemotherapeutic agents produced free radicals in tumor cells, which killed them by capturing electrons and delivered them to oxygen [298]. Moreover, many of these drugs have a large molecular weight, which makes their distribution difficult. This can be exaggerated in abnormal vascular network in hypoxic area within the solid tumors [299]. Hence, drug availability decreases to the levels less than the lethal dose [300]. In addition to this, upregulation of hypoxia-inducible genes involves in chemo-resistance. HIF-1 target hypoxia response elements (HREs) have been found in genes encoding members of the ABC transporters such as MDR1 and BCRP, which their products actively extrude cytotoxic drug out of tumor cells [300, 301]. Hypoxia, also triggers the inhibition of DNA damage and decreased tumor cell senescence particularly in a HIF-1 dependent fashion. For instance, hypoxia inhibits etoposide-induced DNA damage [301]. Cell cycle regulation is an important determinant of tumor resistance [302]. Hypoxic tumor cells, especially being distant from blood supply, have downregulated cell cycle, which protects them against a variety of chemotherapeutic drugs targeted cellular DNA of dividing cells. Such cells also promote drug resistance by altering metabolism and create acidic conditions as previously described [303]. Furthermore, hypoxia, considered as a main driver of autophagy, which have been accompanied with anti-cancer therapeutic resistance. During autophagy, damaged cellular components degraded and tumor cell protected against apoptosis induced by chemotherapy and radiotherapy [304, 305].

Radiation therapy causes cell death by inducing DNA damage, which in the presence of oxygen leads to the generation of free radicals, failure to repair the damage and therefore becomes irreversible. While in hypoxic conditions, the ability of DNA repair increased, results in cell survival and remaining subpopulations responsible for poor outcome [306]. Evidence, also suggested that hypoxia might protect the CSCs to escape from lethal effects of radiotherapy [303]. In addition, radiation can induce transient oxygen fluctuation within the tumor, which leads to ROS generation and HIF-1 expression that in turn promote production of cytokines, VEGF and basic fibroblast growth factor (bFGF). They can eventually, prevent endothelial apoptosis, which provide an additional mechanism for radio-resistance [307]. Several strategies have been exploited to limit hypoxia-induced radio-resistance, such as increase oxygen availability through enhancement of blood flow, mimicking oxygen and targeting hypoxic tumor cells [301].

### Modifying the exosome content in hypoxic tumor microenvironment

Hypoxia triggers exosomes secretion by tumor cells, however, increased exosomes secretion under hypoxic conditions is not a particular feature of tumor microenvironment. Exosomes can change the phenotype of adjacent normal cells and other cells in the tumor microenvironment as well as distant cells through systemic circulation promoting the formation of distant premetastatic niches [308]. Several studies have shown increased amount of special exosome secretion in lung, glioblastoma, prostate cancer, ovarian cancer and breast cancer cell lines through HIF-1α in hypoxic tumor environment [309]. Moreover, it has been shown that hypoxia changed the size of exosomes in pancreatic cancer cell lines [310]. The capability of exosomes to facilitate intercellular communication and their ability to transport various exogenous cargo results in tumor survival, aggressiveness angiogenesis and metastasis. Modifying the content and biogenesis of exosomes in hypoxic tumor microenvironment may be an effective therapeutic intervention approach. Biological feature of exosomes, including their stability, small size, lack of toxicity, communication with other cells, slow clearance from circulation and cargo loading capacity make them an attractive tool for the delivery of therapeutic molecules [309]. An important therapeutic intervention strategy may be to alter the content and biogenesis of exosomes in a hypoxic tumor microenvironnement. The biological characteristics of exosomes such as their stability, tiny size, non-toxicity, interaction with other cells, slow removal of cargo loading capability and circulation, make them an excellent factor for the providing therapeutic molecules [309].

## Treatments of cancer emphasize microenvironment hypoxia in cancer stem cells

CSCs endowed with stemness and tumorigenic properties allow tumor development, metastasis and recurrence. Conventional anticancer approaches mainly target the bulk population of the tumor, whilst sparing CSCs. Moreover, surgery-generated hypoxia is also reported to result in dedifferentiation of tumor cells into tumor-initiating stem-like cells [311]. Therefore, a number of therapeutic approaches in order to kill CSCs have been developed based on altering the microenvironment (niches) protecting them.

In this regard, disruption of vasculature, targeting metabolism and special pH environment represent as alluring landscapes, which can be affected by hypoxia. The strategies directed to target VEGF can lead to interrupt the CSC niche. Several studies showed that bevacizumab significantly reduces the GCSCs in glioblastoma models [312, 313]. A combination of a VEGFR2 antibody with chemotherapy reported by Folkins and coworkers, to be more effective against glioma than chemotherapy alone [314].

Inhibiting the enzymatic pathways and lactic acid generation, as the main part of reprogrammed metabolic network, also presents promising approaches to CSCs elimination. Cui et al. [315], indicated that chronic stress via β_2_-adrenergic receptor upregulated the LDH-A, leading to a switch to lactate formation, in turn promoted stem cell phenotype through USP28/MYC/SLUG axis in breast cancer. And that vitamin C reversed the induction of stem-like phenotype by inhibition of stress-induced LDH-A, and suppressing the lactic acid generation and the USP28/MYC/SLUG pathway in BCSCs. Further, combining of vitamin C, as inhibitor of glycolysis, with the FDA-approved antibiotic doxycycline that reduces cellular respiration, reported to be effective for eradicating CSCs [316].

Mesenchymal CSCs are highly resistant and aggressive subpopulation of GCSCs, which can be resisted and even upregulated after radiation treatment. This subtype of CSCs overexpress ALDH1A3, thus inhibition of ALDH-mediated pathways could be effective in eradication of them [317]. Zhou et al. [318], showed that combining of chemotherapy, together with inhibition of glycolysis by 3-bromopyruvate, effectively eradicates GCSCs in their hypoxic niches, where they exhibit resistant to mono-therapy by chemotherapeutic agents, and tend to reside in hypoxia and perform glycolysis for ATP generation in order to maintain their stemness and highly tumor forming capacity. In addition, in several cancer models, inhibition of PDKII by dichloroacetate has been demonstrated to reverse the metabolic shift from glycolysis to OXPHOS, increase ROS and promote apoptosis in CSCs and tumor cells [319, 320].

Elimination of the stemness characteristics, such as overexpressed drug transporters, considered as the major purpose to improve efficacy of chemotherapy. The combination therapy consists of inhibitors of HIFα or HIF target gene (such as ABC transporters) suggested using concomitantly with chemotherapeutic agents. For instance, anti-MDR1 siRNA (siMDR1) successfully improved the chemo-sensitivity of human colon CSCs [321]. Several tyrosine kinase inhibitors such as imatinib, erlotinib, nilotinib and lapatinib have been applied to inhibit ABC transporters [322]. In addition, various strategies based on suppression of transporter function, including nanoparticle-mediated delivery of inhibitors, competitive and allosteric modulators, regulator of transcriptional and signaling pathways involving ABC transporters have been tested to overcome chemo-resistance in CSCs [323], however, none of them has been approved yet.

In hypoxiamediated CSC resistance, several signaling pathways recognized to be involved. Therefore, these pathways draw much attention as potential targets to eliminate CSCs, particularly in hypoxic TME. Hypoxia promotes Notch and Wnt/β-catenin signaling to CSC formation and maintenance, mainly in a HIF-1α-dependent manner [324]. Targeting Notch4 could decrease the number and activity of BCSC in vivo, as well as in vitro [325]. Zhou and coworkers [326], recently showed that Notch4 promotes EMT and quiescence in mesenchymal BCSC via activation of SLUG and GAS1, suggesting Notch4-SLUG-GAS1 serves as a candidate for tumor treatment by elimination of mesenchymal BCSC stemness and overcoming the lethal form of chemo-resistant and metastatic triple-negative breast cancer. Seo and colleagues [327], further showed that hypoxia-induced Notch signaling increases self-renewal, drug resistance, sphere formation and expression of genes relating to CSC features, such as SOX2, ALDH, and ABC transporters in ovarian CSCs (OCSCs). In fact, Notch signaling mediates the hypoxia-induced expression of Sox2 in a HIF-1 dependent manner, and Sox2 in turn upregulates the ABCB1 and ABCG2, thereby promoting drug resistance, as well as triggering sphere formation and CSCs features. They also showed that the inhibition of Sox2 in a HIF-1 abrogates hypoxia-induced CSC features. Their finding proposed the hypoxia- Notch1-SOX2 circuit as a potential target to eradicate OCSCs in hypoxic condition. Moreover, suppression of MMP-2, 9 and VEGF abrogates Notch signaling in hepatocellular carcinoma cells and decreases tumor invasiveness [328]. Hypoxia triggers phenotypic plasticity of CSCs by activation of Wnt signaling. Yan et al. [329], also indicated that HIF-1α expression increased during acute hypoxia (6–12 h), whereas, HIF-2α overexpression continuously occurred in chronic hypoxia (48 h) and activated Wnt and Notch signaling and expression of stem cell markers Nanog, c-Myc, and Oct4. Thereby, HIF-2α promoted stem phenotype conversion, augmented tumorigenesis and induced resistance to chemotherapeutic drug paclitaxel. Hypoxia, also, upregulates β-catenin transcription, downstream of Wnt, via stabilization of HIF-1α in leukemia CSCs, and promotes their survival [330]. Different drugs aimed at inhibiting the Wnt signaling pathway have been used to impede this pathway in CSCs [328, 331]. Evodiamine (Evo), an inhibitor of the Wnt signaling, demonstrated that downregulated the expression of Sox2, KLF4, Bmi1 and Oct4 in GCCs, thus promoted apoptosis and decreased proliferation, sphere formation capacity and resistance to oxaliplatin. Evo, also, downregulated the expression of Slug, Twist, Zeb1 and vimentin, and thereby acquired the inhibitory effect on EMT. Further, Evo reduced cMyc, cyclin D1, as well as βcatenin expression in spheroids from GCCs in gastric cancer [332]. Trifluoperazine is another inhibitor of the Wnt/β-catenin pathway with potential anti-CSC properties, which suppressed the CSC marker (CD44/CD133) expression and spheroid formation capacity of LCSCs. Combining of this inhibitor with gefitinib or cisplatin diminished chemo-resistance of lung cancer [333]. Natural products have been also identified by similar effect. Curcumin inhibits β-catenin nuclear localization, hampers the Slug activation and reverses suppressed E-cadherin expression, subsequently inhibits EMT and migration of BCSCs [334]. Cucurbitacin B, inhibitor of Wnt/β-catenin signaling derived from pumpkins and guards, inhibits stemness and metastasis of non-small cell lung cancer (NSCLC). It has also the anti-angiogenic and anti-invasive potential against metastatic NSCLC [335].

Hedgehog (Hh) signaling, one of the main pathways related to stemness and CSC development, interacts with other critical molecular pathways, in particular Ras/Raf/MEK/Erk, PI3K/Akt/mTOR and Notch. Hence, combined targeting of the component belonged to different signaling pathways result in more effective anti-cancer strategies [336]. Mitogen-activated protein kinase (MAPK)/ERK signaling is one of the key pathways exploited by hypoxia to promote CSC phenotype. In response to chemotherapy, HIF promotes propagation of BCSCs and thereby tumor recurrence. One of the mechanisms proposed is activation of cystine transporter xCT and the glutamate-cysteine ligase by HIF-1α that leads to increased intracellular glutathione, which in turn inhibits mitogen-activated protein kinase MEK activity. Loss of MEK-ERK signaling causes FoxO3-mediated transcriptional activation of Nanog, a pluripotency factor required for propagation of BCSCs. Targeting this HIF-1-regulated pathway might inhibit the BCSC enrichment and tumor relapse after chemotherapy [337].

PI3K/Akt signaling is the other important pathway mediating CSC resistance. It is indicated that ERK1/2 and PI3K/Akt pathways act at downstream HIF-1α signaling in response of GCSCs to hypoxia, and that inhibition of these pathways abrogate propagation of CD133-expressing GCSCs [73]. Recent finding implicated Akt/Notch1 signaling cascade as promoter of stemness and pancreatic CSCs (PCSCs)-associated resistance, which exacerbated in collaborate with hypoxia. Blockade of this pathway augmented the cytotoxic effect of gemcitabine [338]. In hypoxic GCSCs, HIF2-α through activation of Akt and Erk1/2 pathways, upregulates prostatic acid phosphatase (PAP) to produce adenosine, which binds to adenosine receptor A2B, thereby increases proliferation and tumorigenic capacity [339]. Recently, it is demonstrated that celastrol, an anti-glioma medicine, downregulated the HIF-1α, blocked the PI3K/Akt/mTOR signaling and disrupted angiogenesis and VM formation. [340]. mTOR, a downstream mediator in PI3K/AKT pathway, complicatedly regulated by HIF-1α signaling and acts as a nutrient/hypoxia sensor to modulate protein synthesis. Marhold et al. [341], identified that mTOR was deactivated in response to HIF1α upregulation, and inhibits proliferation, but interestingly promotes quiescence and survival of PCSCs in the hypoxic niche. Its possible mechanism involves the activation of Akt signaling through mTOR/S6K/IRS-1 feedback loop. This might be an explanation for the failure of selective mTOR inhibitors in clinical trials. Altogether, given the complex regulation of the molecular pathways and their interactions in response to HIFs in CSCs, we found that targeting of several signaling molecule of these pathways provides more efficient approaches to overcome CSCs-associated resistance in hypoxia.

## Conclusion and future prospectus

While hypoxia has been emerged as a key player in allowing tumor survival, dissemination and invasiveness, this review summarizes the significance of HIFs in CSC development, malignant progression and cancer resistance to chemo-, radio- and immuno-therapy. As a result, HIF stabilization in hypoxic tumor cells induces the expression of specific target genes encoding proteins that promote neo-angiogenesis (VEGF), metabolic changes (glycolytic enzymes and glucose transporters), stemness, EMT and metastasis (CXCR4, E-cadherin). Further, tumor hypoxia is thought to promote the production of cytokines and chemokines, which recruit pro-tumor immune cell and diminish tumor immune responses. Therefore, better understanding of the hypoxia signaling cascade can open new windows to design strategies for targeting HIFs and management of hypoxic microenvironment, led to (i) limit tumor expansion; (ii) sensitize CSCs; and (iii) overcome tumor resistance and recurrence.

## Abbreviations

ABC: ATP-binding cassette transporter
BCSCs: Breast cancer stem cells
bFGF: Basic fibroblast growth factor
BMDCs: Bone-marrow derived cells
BMI1: Polycomb complex protein BMI-1
CAs: Carbonic anhydrases
CAIX: Carbonic anhydrase 9
CCL28: CC-chemokine ligand 28
CTL: Cytotoxic T, lymphocytes
CXCR4: C–X–C chemokine receptor type 4
ECM: Extracellular matrix
EGF: Epidermal growth factor
EMAPII: Endothelial-monocyte-activating polypeptide II
EMT: Epithelial–mesenchymal transition
FGF2: Fibroblast growth factor 2
GBM: Glioblastoma
GLUT1: Glucose transporter1
Hh: Hedgehog
HAPs: Hypoxia-activated prodrugs
HIF1: Hypoxia-inducible factor 1
IGF-2: Insulin-like growth factor-2
iNOS: Inducible nitric oxide synthase
JAK/STAT: Janus-activated kinase/signal transducer
KLF4: Krupper-like factor 4
LDH-A: Lactate dehydrogenase A
LOX: Lysyl oxidase
MDR1: Multidrug resistance 1 receptor
MDSCs: Myeloid-derived suppressor cells
miRNA: Micro RNA
MMP: Matrix metalloproteinases
Myc: Myelocytomatosis oncogene product
NANOG: Tir nan Og
NE: Neutrophil elastase
NETs: Neutrophil extracellular traps
NF-kB: Nuclear factor-kB
OCT4: Octamer-binding transcription factor
p53: Protein 53
PAI-1: Plasminogen activator inhibitor-1
PDCD1: Programmed cell death 1
PDGF: Platelet-derived growth factor
PDK1: Pyruvate dehydrogenase kinase 1
PD-L1: Programmed cell death ligand-1
PI3K/PTEN: Phosphatidylinositol 3-kinase/phosphatase, tensin homolog
ROS: Reactive oxygen species
SOX2: Sex determining region Y HMG-box 2
TANs: Tumor-associated neutrophils
TGF-β: Transforming growth factor beta
TME: Tumor microenvironment
Tregs: Regulatory T cells
TSPs: Thrombospondins
VEGF: Vascular endothelial growth factor
VEGFR: Vascular endothelial growth factor receptor
Wnt: Wingless
ZEB1: Zinc-finger E-box binding homeobox 1
